# Synergistic CuCoS–PANI materials for binder-free electrodes in asymmetric supercapacitors and oxygen evolution†

**DOI:** 10.1039/d3na01066j

**Published:** 2024-01-26

**Authors:** Haseebul Hassan, Muhammad Waqas Iqbal, Hussein Alrobei, Fareeha Riasat, Amir Muhammad Afzal, Ahmad M. Saeedi, Hasan B. Albargi, Arslan Rehmat

**Affiliations:** a Department of Physics, Riphah International University Campus Lahore Pakistan waqas.iqbal@riphah.edu.pk; b Department of Mechanical Engineering, College of Engineering, Prince Sattam Bin Abdul Aziz University Al-Kharj 11942 Saudi Arabia; c Department of Physics, Faculty of Applied Science, Umm AL-Qura University Makkah 24382 Saudi Arabia; d Department of Physics, Faculty of Science and Arts, Najran University PO Box 1988 Najran 11001 Saudi Arabia; e Department of Physics, Sejong University South Korea

## Abstract

In advanced electronics, supercapacitors (SCs) have received a lot of attention. Nevertheless, it has been shown that different electrode designs that are based on metal sulfides are prone to oxidation, instability, and poor conductance, which severely limits their practical application. We present a very stable, free-standing copper–cobalt sulfide doped with polyaniline as an electrode coated on nickel foam (CuCoS/PANI). The lightweight nickel foam encourages current collection as well as serving as a flexible support. The CuCoS–PANI electrode had a substantially greater 1659 C g^−1^ capacity at 1.0 A g^−1^. The asymmetric supercapacitor (ASC) can provide an impressive 54 W h kg^−1^ energy density while maintaining 1150 W kg^−1^ power. Additionally, when employed as an electrocatalyst in the oxygen evolution reaction, CuCoS/PANI exhibited a 200 mV overpotential and 55 mV dec^−1^ Tafel slope, demonstrating its effectiveness in facilitating the reaction.

## Abstract 2

In advanced electronics, supercapacitors (SCs) have received a lot of attention.

## Graphical abstract

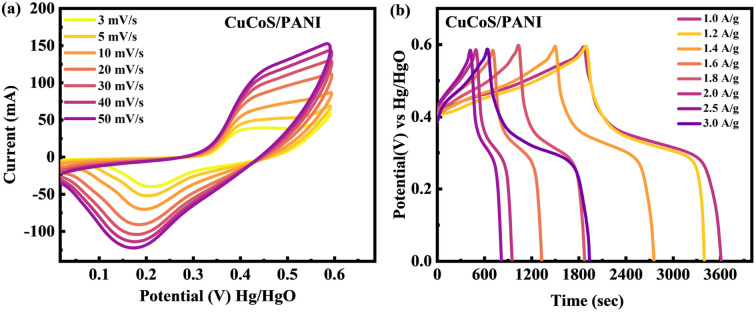

## 1. Introduction

The continuous need for energy driven by a growing world population and industrialization is causing fossil fuel supplies to be depleted quickly.^1^ So renewable green energy is an urgent need in this era. These renewable energy resources such as sun or wind energy require a storage system alongside them to operate adequately.^2,3^ Currently, batteries and capacitors are widely adopted energy storage devices.^4,5^ The advancements in capacitors resulted in the development of supercapacitors (SCs) that exhibit high energy and power densities thus enabling them to bridge the gap between conventional capacitors and batteries. SCs because of their excellent mechanical stability, long-term stability, safe operation mode, and environmental friendliness are becoming more popular.^6–9^ The selection of the electrode, electrolytes, and assembly method has a big impact on the properties of supercapacitors (SCs).^10^ Notably, the electrode design has gained significant attention as a crucial area of research in recent times.^11,12^ Currently, scientists are extensively researching transition metal oxides (TMOs), transition metal sulfides (TMSs), and transition metal phosphates (TMPs) for this purpose.^13–15^

Numerous strategies have been proposed to increase the supercapacitor energy density. The two main methodologies used in research can be stated as follows: (1) assembling an asymmetric supercapacitor, and (2) designing an electrode that possesses greater specific capacity. High specific capacity and strong cycle stability can be achieved by any technique through meticulously selecting a suitable electrode material and implementing the appropriate nanostructure design.^16,17^ Metal sulfides are considered well-known electrodes for SCs and LIBs that possess exceptional chemical stability, abundant valence states, stronger crystal lattice, and enhanced electrical conductivity.^18,19^ The band gap, electronegativity, and conductivity of metallic sulfide samples are often higher than those of their corresponding oxide counterparts.^20–22^ Recently, metal sulfides (MSs) like CuS, Co_9_S_8_, MoS_2_, FeS_2_, MnS_2_, and Ni_3_S_2_ have received a lot of attention as viable alternative pseudocapacitive electrode materials in SCs because of their increased specific capacity, energy density, and power density.^23,24^ One intriguing possibility for SC electrode materials is to merge two metal sulfides, which exhibits a superior theoretical specific capacity because of the enhancement of mass transport, ion diffusion, and electron transfer.^25,26^ Binary metal sulfides exhibit richer redox reactions and greater electronic conductivity than their single-component counterparts, leading to improved electrochemical performances.^27,28^ Jinxue Guo *et al.*^29^ prepared CuS double-shell hollow nanocages from copper nanotubes, which exhibit large specific capacity, good cycling stability, and outstanding rate performance. Jing Zhang *et al.*^30^ prepared 3D hierarchical CuS microspheres using a solvothermal approach which delivered an energy density of 15.06 W h kg^−1^. Jiangfeng Li *et al.*^31^ reported 366 F g^−1^ specific capacity with porous nanoflake of CoS. The CoS can retain 95% capacity after 1300 cycles.

The analogous conducting polymer polyaniline (PANI) is also recognized as one of the most alluring electromagnetic interference (EMI) protecting materials among the wide variety of materials used in supercapacitors. PANI has numerous advantages, including high electrical conductivity, low weight, high stability, and simple synthesis. However, PANI tends to expand and contract throughout the charging and discharging process due to its small potential window in aqueous electrolytes, resulting in poor cyclic stability for supercapacitors as well as low energy density. Consequently, combining PANI with metal sulfides is a beneficial strategy for improving electrochemical performance and attaining long-cycle stability in capacitors.^32,33^ In addition, a single electron pair of the N atoms can establish an N–metal bond with the metal sulfides (MSs).^34^ In particular, sulfides may operate as strong electron carriers, allowing them to produce permeable conductive paths through the cooperation of PANI polymer chains, an increase in the efficiency of charge exchange, and extent of stability over redox cycles.

In this research we synthesized a copper–cobalt sulfide doped with polyaniline (PANI) on a nickel foam (CuCoS–PANI) to check its electrochemical performance. Nickel foam (NF) is an interesting flexible substrate material because of its availability, high electrical conductivity, and superior mechanical properties. Furthermore, NF can bear significant mechanical stress while being easily coiled, braided, and folded. For these reasons, it is a promising material for fabricating soft circuits with high mechanical characteristics and flexibility. Our objective is to develop a highly efficient, powerful, and eco-friendly asymmetric energy storage system. To our knowledge, the deposition of CuCoS–PANI on NF for supercapacitor (SCs) applications remains unexplored in existing literature, highlighting the novelty of our work. The electrochemical parameters were checked in both half and full-cell structure. First, the binary metallic copper–cobalt sulfide doped with PANI was assessed in a three-electrode assembly with Hg/HgO as the reference electrode and platinum wire as the counter electrode.

Secondly, we built an asymmetric supercapacitor by using activated carbon as the negative electrode and CuCoS/PANI as the positive electrode in a hybrid device we designed. To assess its durability, the device underwent 12 500 cycles of charging and discharging during the testing process. Thirdly, the potential of CuCoS/PANI in the oxygen evolution reaction was also determined.

## 2. Experimental

### 2.1. Materials

The materials used in the present investigation were of analytical grade and were utilized precisely as received. Thermo Fisher Scientific provided KOH, hydrochloric acid (HCl), carbon black, copper nitrate (Cu(NO_3_)_2_·2H_2_O), cobalt(ii) nitrate (Co(NO_3_)_2_·6H_2_O), activated carbon, and sodium sulfide (Na_2_S). Sigma-Aldrich supplied us with nickel foam for our research. We acquired a counter electrode (platinum wire) and reference electrodes (Hg/HgO) from ALS Co. Ltd (Japan) to complete a three-electrode assembly. During synthesis, nickel foam (1 cm × 3 cm) was repeatedly submerged in acetone, a solution of HCl, and ethanol in order to eliminate the oxide layer and impurities from the surface, and then air-dried.

### 2.2. Material synthesis

0.4 mL of hydrochloric acid (HCl) was added to 100 mL of deionized water (DIW), and the mixture was put on a magnetic stirrer. Subsequently, 4 mL of aniline was added, resulting in the formation of a solution with a vibrant orange color. It was stirred for 30 min and its temperature maintained between 0 °C and 5 °C by using ice pieces. Then the second solution was made by adding 5 g of ammonium persulfate (APS) in 50 mL of DLE. Both the solutions were mixed dropwise. The whole solution was stirred for 1 h. A dark colored solution was formed. Then it was filtered and dried at a temperature of 60 °C for 6 h.

Cobalt sulfide was synthesized using a conventional hydrothermal method similar to that reported by A. Mindil *et al.*^35^ This kind of approach stands out as the most feasible approach for synthesizing nanoparticles. Moreover, the recommended temperature range for this technique is from 140 °C to 300 °C, which corresponds to the boiling point of the great majority of the material. Cobalt sulfide nanoparticles have been synthesized *via* the conventional hydrothermal technique. At a temperature of 60 °C, a solution containing 9.122 g (0.8 M) of cobalt(ii) nitrate dissolved in 30 mL of DIW was added and stirred for 30 min. Then, at a temperature of 60 °C, 12 g (0.8 M) of sodium sulfide hydrate was mixed with 40 mL of DIW. The two solutions were then gently added dropwise while stirring for 2 h. The process involved transferring a combination of the precursor materials and the sulfide precursor to the hydrothermal reactor and maintaining it there for 7 h at 140 °C. The precipitate was then collected and centrifuged at a speed of 6000 rpm. In the end, the black precipitate was dried for 7 h at 60 °C.

The procedure involved the preparation of a 0.8 M solution of copper(ii) nitrate (7.13 g) in DIW (40 mL). Then, the solution temperature was raised to 60 °C and it was stirred for 30 min. A second solution was made with 0.8 M sodium sulfide hydrate (12 g) in deionized water (40 mL). The mixture was slowly added dropwise to the copper(ii) nitrate solution while maintaining a temperature of 80 °C. The reaction mixture was continuously stirred for 1 h to ensure thorough mixing and reaction. The development and growth of CuS particles were aided by this mechanism. The sterilized solution was then moved to a Teflon-coated autoclave that was subsequently heated to 140 °C and kept there for 7 h. This prolonged hydrothermal treatment allowed for the complete transformation of the reaction mixture into copper sulfide. Following the hydrothermal synthesis, the resulting copper sulfide product can be collected, washed, and characterized for further analysis or application in various fields.

**Fig. 1:**
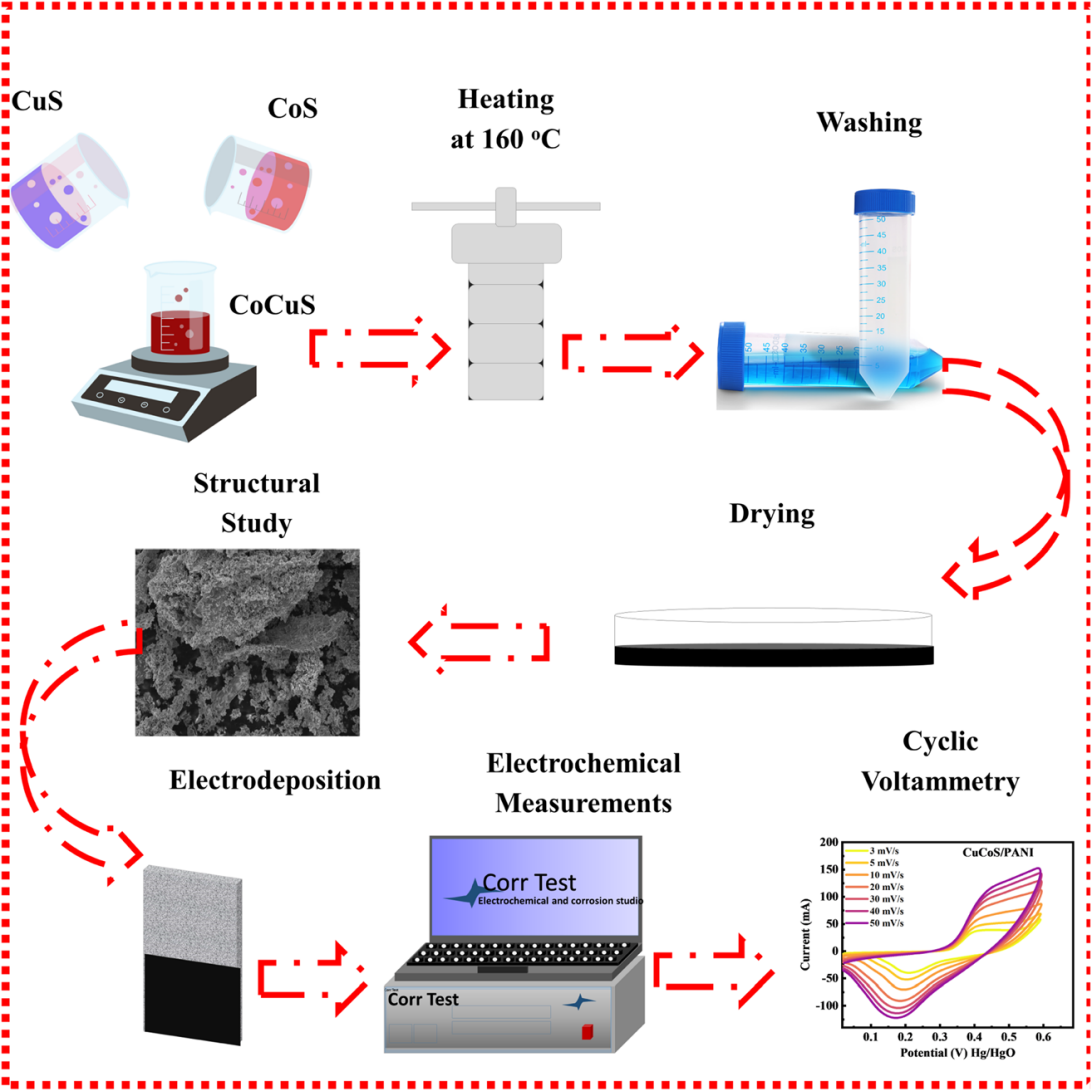
Systematic approach to synthesize CuCoS through a hydrothermal technique.

The hydrothermal method was used to effectively synthesize CuCoS with an ideal weight ratio of 50/50. Na_2_S·9H_2_O, Co(NO_3_)_2_·6H_2_O, and Cu(NO_3_)_2_·2H_2_O were dissolved in 25 mL of DIW. 0.4 M cobalt nitrate was added to 40 mL of DIW and stirred for 30 min at 60 °C. Then, either 0.8 M sodium sulfide or 0.4 M copper nitrate was mixed with 30 mL of DIW and added dropwise at 80 °C while stirring for 1 h. The solution, which produced a black precipitate of CuCoS, was heated in an oven at 140 °C for 7 h. This whole mixture was cooled to room temperature. A similar method which is described in the above section was followed to obtain the final product. PANI was then incorporated into the heterostructure by simply grinding in the best weight ratio 10/90% as reported earlier in our work.^35^ CuCoS/PANI was synthesised using a step-by-step process, which is shown in Fig. 1.

### 2.3. Electrochemical testing

By using cyclic voltammetry (CV), galvanostatic charge–discharge (GCD), and electrochemical impedance spectroscopy (EIS), the electro-active characteristics of the synthesized CuS, CoS, and CuCoS materials were examined. These measurements were performed in a 2 M potassium hydroxide electrolyte with the help of a CS-300 workstation. A Pt wire and Hg/HgO were used in a conventional half-cell arrangement for the electrochemical evaluation of the electrodes. This configuration allows the comparison and evaluation of electrode performance. In the present research, the working material CuCoS/PANI was deposited onto an NF substrate using an electrodeposition approach. To begin with, an initial solution was produced by combining 0.8 M CuCoS/PANI, which contains aniline polymers, copper, cobalt, and sulfur ions. The CuCoS/PANI was developed on a 1 × 1 cm^2^ area of 3 × 1 cm^2^ NF. NF contained 6.5 mg of the active substance.

The charge was balanced using eqn (1).1
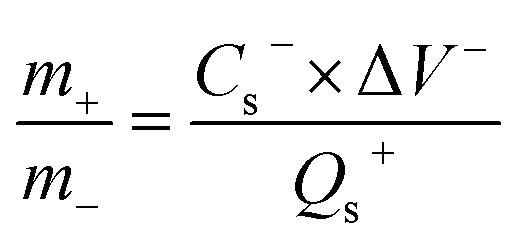


Activated carbon (AC) served as the cathode and CuCoS/PANI as the anode in asymmetric design. The stability of the CuCoS/PANI//AC device was investigated by offering 12 500 cycles. Conductivity measurements were also performed with the electrolyte and electrode which is presented in the ESI section.†

## 3. Results analysis

### 3.1. Structural characteristics

**Fig. 2:**
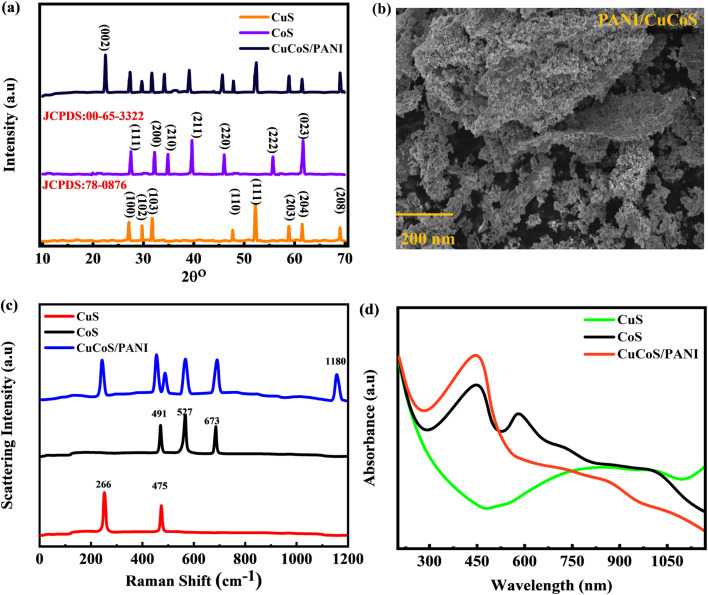
(a) XRD analysis for CuS, CoS, and CuCoS/PANI. (b) SEM image for CuCoS/PANI. (c) Raman Spectrum for CuS, CoS, and CuCoS/PANI. (d) UV-vis spectrum for CuS, CoS, and CuCoS/PANI.

X-ray diffraction (XRD) analysis of CuCoS provides valuable information about the crystal structure and phase identification of the material (Fig. 2(a)). The crystal structure may be determined by comparing the XRD pattern diffraction peaks to those listed in the JCPDS (Joint Committee on Powder Diffraction Standards) card database. In the experimental apparatus, Cu K radiation with a wavelength of 1.5406 was utilized. With a step size of 0.02° and a scan pace of 2° min^−1^, the 2*θ* scanning range extended from 10° to 80°. The obtained XRD pattern indicates distinct diffraction peaks at different 2*θ* angles. The diffraction peak configurations of purified CuS nanorods correspond well with JCPDS no. 78-0876 for the hexagonal crystal plane system of face-centered cubic (fcc) CuS nanoparticles. CuS nanorods exhibit prominent indexing peaks with corresponding reference planes at 28.65°(100), 29.5°(102), 32.2°(103), 48.2°(110), 52.2°(111), 59.3°(202), 60.7°(204) and 69.5°(208) respectively.^36^ The CoS XRD maxima occur at the 2*θ* of 28.8°, 33.76°, 35.69°, 41.1°, 45.8°, 56.3°, and 61.2° which correspond to the (111), (200), (210), (211), (220), (222), and (023) planes (JCPDS: 00-65-3322-CoS_2_).^37,38^ The XRD analysis provided crucial insights into the crystallographic properties of CuCoS, thereby facilitating the characterization of this material for our research. The formation of CuCoS is verified by XRD, which exhibits a pattern that is equivalent to the peaks of CoS and CuS. The hump around 24° belongs to the (200) plane, indicating the presence of PANI. The size of the nanoparticles was estimated from Scherrer's formula.^39^2
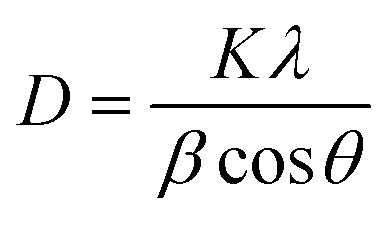


In eqn (2), the crystal size is represented by *D*, the wavelength of the incident X-ray is denoted by *λ*, *K* represents a constant, and *θ* signifies the angle of diffraction. By applying Scherrer's formula, we determined that the crystal size of CuCoS was approximately 59 nm, indicating the average particle size of CuCoS in the composite. Similarly, the crystal size of PANI was measured to be approximately 55 nm, which corresponds to the size of every PANI particle. Particularly, the CuCoS/PANI composite exhibited a crystal size of approximately 65 nm, indicating a modest increase in the average particle size as a consequence of the incorporation of CuCoS into the PANI matrix.

Fig. 2(b) depicts the CuCoS/PANI composite morphology as recognized through a scanning electron microscope (SEM) image. The image reveals the composite surface characteristics, which conspicuously display the presence of numerous nanoparticles. These nanoparticles are dispersed over the surface, producing a distinct pattern and contributing to the overall structure of the CuCoS/PANI composite. The coexistence of CuCoS nanoparticles and PANI in an intriguing configuration resembles small nanostructures. The figure highlights a beneficial relationship between the two constituents of the composite by showing the strong bond between CuCoS particles and PANI, which generates intricate networks of connected structures. These nanostructures suggest that CuCoS and PANI are often combined to form the composite, which may result in a rise in conductance and SSA. The SEM images for CuS and CoS are presented in Fig. S2.†

The CuS, CoS, and CuCoS/PANI compound were then subjected to a Raman study to determine the vibrational states (Fig. 2(c)). The Raman spectra for CuS exhibit two distinct strong peaks, one at 475 cm^−1^ and the other at about 266 cm^−1^.^40^ The peak observed in the region of shorter wavelengths corresponds to the CuS vibrational mode, revealing the particular vibrational patterns associated with this material. In contrast, the peak observed in the longer wavelength region is attributed to the S–S stretching vibration mode of the lattice, which represents the hexagonal CuS structure. The results we obtained indicate that crystalline copper sulfide was effectively produced throughout the synthesis procedure. The Raman spectrum obtained from the CoS sample demonstrated the characteristic of cobalt sulfide. Three peaks that were very prominent at 491 cm^−1^, 527 cm^−1^, and 673 cm^−1^ were typically assigned to vibrational states E_1g_, E_2g_, and A_1g_, respectively.^41^ The CoS vibrational mode, connected with the peak in the shorter wavelength area, reveals details of the bonding properties of the materials. The Raman spectrum of CuCoS/PANI shows similar peaks to CuS and CoS. In addition, the spikes present at 1180 cm^−1^ confirm the presence of PANI.^42^

**Fig. 3:**
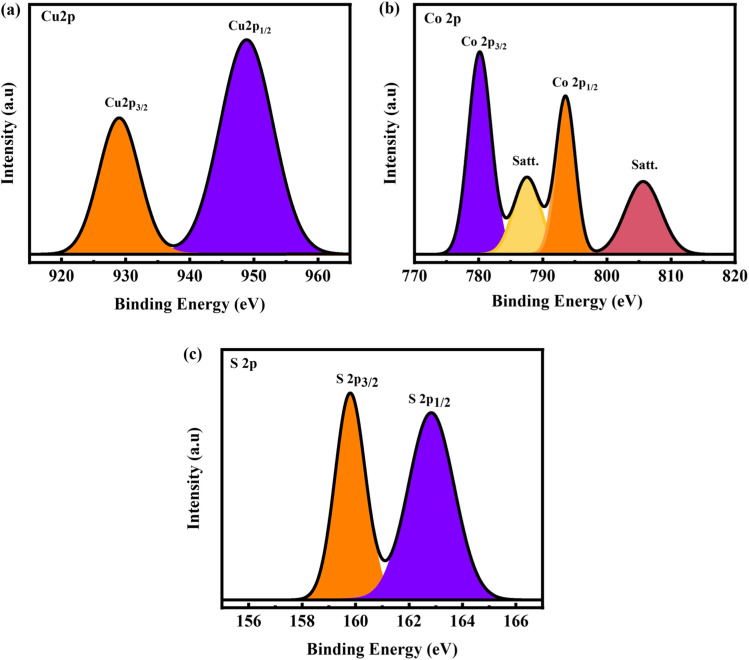
(a–c) XPS spectrum for Cu 2p, Co 2p, and S 2p, respectively.

UV spectroscopy is a powerful tool for investigating the electrical and optical absorption characteristics of various materials. The band gap energy and potential usefulness of CuCoS/PANI as a supercapacitor were determined by examining its UV-visible absorption spectra (Fig. 3(d)). The spectrum absorption peaks and edge position can indicate the electronic transitions occurring within a material. The UV investigation of CuCoS/PANI provides valuable information regarding its bandgap energy, optical properties, and light absorption potential. Fig. 2(d) shows the UV analysis for all three samples. CuS has two different broader characteristics in its absorption spectra. The peak at 452 nm indicates the visible spectrum.^43^ The peak at 350 nm is due to CoS.^44^ The UV spectrum shows the presence of CuS and CoS.

The chemical states and composition of CuCoS were checked through X-ray photoelectron spectroscopy (XPS). The main goal of the XPS investigation was to determine the Cu, Co, and S oxidation states in the as-prepared bulk CuS material. The core level spectra of Cu 2p in high resolution XPS are shown in Fig. 3(a), respectively. Two different binding energies, at 929.4 eV and 950.4 eV, corresponding to the Cu 2p_3/2_ and 2p_1/2_ states, were seen in the high-resolution Cu 2p spectra of CuS.^45^ Co 2p high-resolution spectra were used in the XPS study as well. Two peaks, at 780.5 eV and 793.5 eV, were identified as reflecting the Co 2p_3/2_ and Co 2p_1/2_ states, respectively, after the Co 2p spectra were deconvoluted into four peaks (Fig. 3(b)). Furthermore, two additional maxima, at 789 eV and 806 eV, were detected, both of which are characteristic of CoS materials. The presence of Co^2+^ species is suggested by the satellite peaks.^46^ The XPS spectra collectively indicate the effective fabrication of CoS. The high-resolution S 2p spectra also showed three distinct peaks. Spectral Gaussian fitting revealed that CuS has many distinctly shaped S sites. S^2−^ is responsible for the signals seen at 159.8 (S 2p_3/2_) and 162.7 eV (S 2p_1/2_) (Fig. 3(c)). These results verify the existence of several sulfur species in the CuCoS material.^47^ The binding energies and peaks that have been found are consistent with those that have been reported before, proving the materials to be composed of CuS and CoS. These results contribute to a comprehensive comprehension of the elemental composition of the synthesized materials, which is essential for further characterizing their electronic and chemical behavior.

**Fig. 4:**
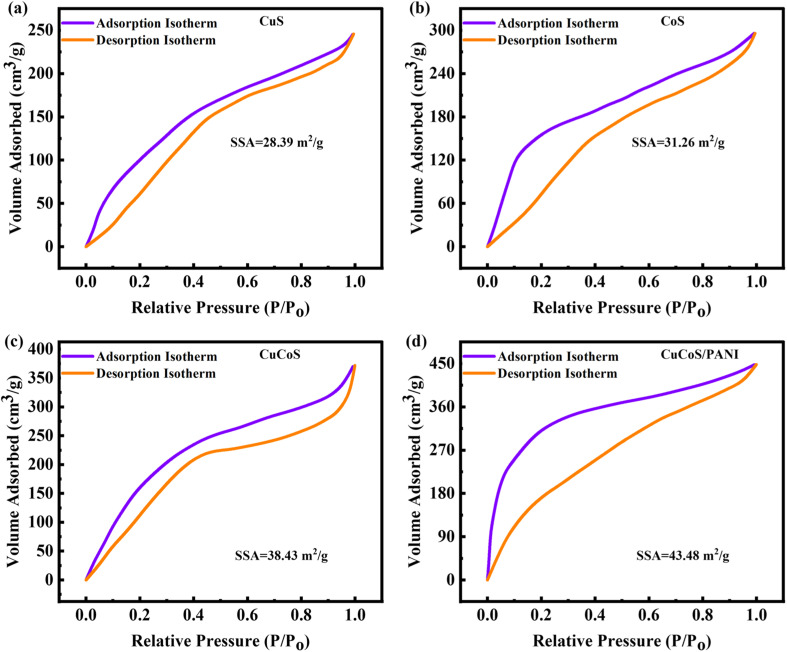
(a–d) BET isotherms for CuS, CoS, CuCoS, and CuCoS/PANI, respectively.

The BET (Brunauer–Emmett–Teller) technique is commonly used for calculating specific surface area. Since the BET technique is recognized as a conventional method, the goal of this research is to identify the particular area of the surface of the CuS, CoS, CuCoS, and CuCoS/PANI using this method (Fig. 4). The surface area is significantly enhanced by mesopores and micropores.

The surface area of CuS, CoS, CuCoS and CuCoS/PANI nanocomposite materials is estimated to be 28, 31, 38, and 43 m^2^ g^−1^. Incorporating Cu nanoparticles into the resulting nanocomposite significantly increased the surface area of CoS. Additionally, the BET surface area is also increased with PANI addition. The BET graph of CuCoS/PANI demonstrated a type-IV form, showing its particular adsorption behavior. Notably, the inclusion of a small hysteresis loop in the graph offered convincing proof of the open porous structure of CuCoS/PANI. For possible uses in gas adsorption, catalysis, and energy storage devices that profit from readily available and well defined porous materials, this characteristic open porosity is significant. The diversity in pore sizes and volumes across these materials could impact ion diffusion kinetics and storage capabilities, crucial factors in determining the rate capability and cycling stability in electrochemical applications. Specifically, smaller and more evenly distributed pores tend to promote faster ion transport, potentially leading to improved rate capability, while larger pore volumes might contribute to greater ion storage capacity. The SSA, pore size, and pore volume for CuS, CoS, CuCoS, and CuCoS/PANI are shown in Table 1.

**Table 1:** Surface area, pore size, and pore volume for CoS, CuS, and CuCoS through BET measurements

Materials	Surface area (m^2^ g^−1^)	Pore volume (cm^3^ g^−1^)	Pore size (nm)
CoS	28 m^2^ g^−1^	0.028	0.12
CuS	31 m^2^ g^−1^	0.043	0.18
CuCoS	38 m^2^ g^−1^	0.056	0.27
CuCoS/PANI	43 m^2^ g^−1^	0.068	0.33

### 3.2. Electrochemical measurements

**Fig. 5:**
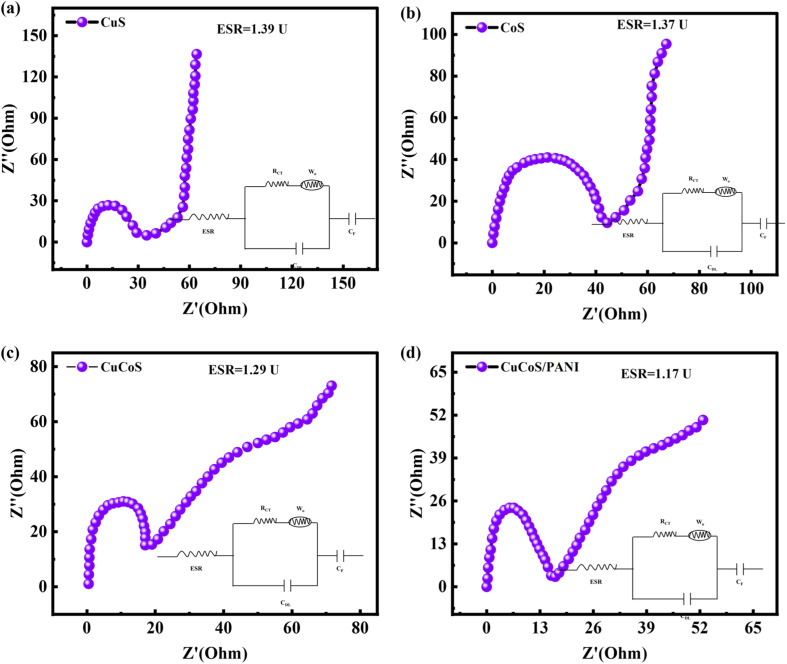
(a–d) EIS spectrum for CuS, CoS, CuCoS, and CuCoS/PANI, respectively.

One of the most used analytical techniques for characterizing supercapacitors is electrochemical impedance spectroscopy (EIS). EIS had been set up with a measuring range of 0.1 kHz to 100 kHz at an open circuit potential and the results are evaluated with Nyquist plots. The EIS test for CoS, CuS, CuCoS, and CuCoS/PANI is shown in Fig. 5(a)–(d). The transfer of charge resistance was responsible for the semi-circular form in the EIS spectrum. The intersection of the real line and the high-frequency line provides a convenient measurement of the internal resistance. The EIS graphs were linear. The imaginary portion continually declines with falling frequency. Faradaic reactions along with the double-layer capacity were demonstrated by the appearance of a semicircle. Faraday interface charge transfer resistance (*R*_ct_) is represented by the high-frequency diameter of the semicircle, and typical Warburg resistance (*W*_0_) is represented by the low-frequency slope of the straight line.

At lower potentials, the electrochemical impedance spectroscopy (EIS) graph exhibited a semicircular shape, while at higher potentials, it transformed into a linear form. The Equivalent Series Resistance (ESR) is determined by adding the ionic resistance, inherent resistance, and contact resistance. The ESR values for CuS, CoS, CuCoS and CuCoS/PANI were 1.39, 1.37, 1.29 and 1.17 U, respectively. The incorporation of PANI into the CuCoS nanocomposite significantly reduced the ESR.

**Fig. 6:**
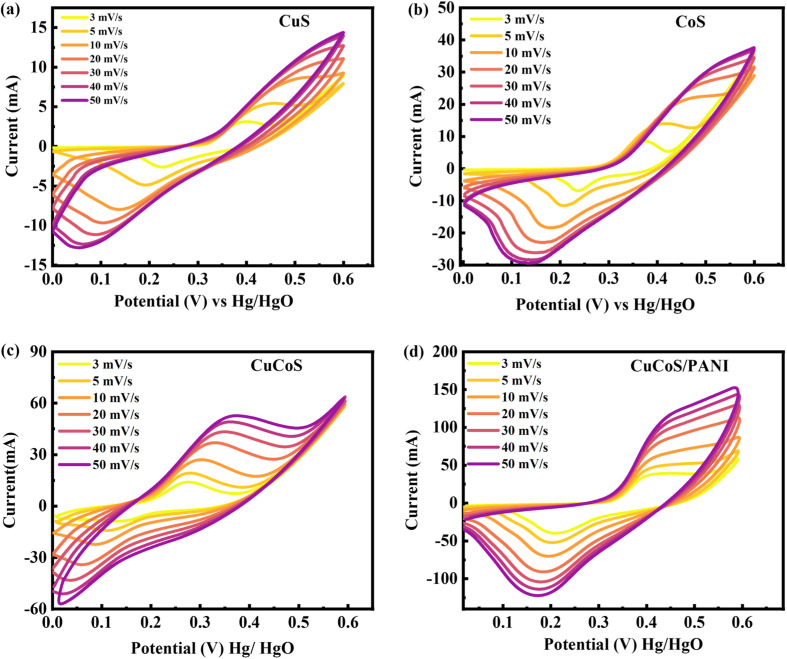
(a–d) CV tests for CuS, CoS, CuCoS, and CuCoS/PANI.

To investigate the process of reduction and oxidation, cyclic voltammetry (CV), an effective and efficient electrochemical technique, is frequently used. The CV curves were not rectangular; rather, redox processes caused oxidation/reduction spikes. Fig. 6(a)–(d) illustrate the CV graph for CuS, CoS, CuCoS, and CuCoS/PANI at the O.P. ranges of 0–0.6 V with scan speeds of 3–50 mV s^−1^. CV patterns of the same sample may differ depending on the shape and surface qualities of the produced electrodes, as has been recently reported. The symmetry of the CV tests was used to characterize the properties of the materials. The capacitive storage capacity of the electrodes was also evaluated using CV plots. The liquid ultimately contained far fewer electrons than the anode at a negative potential. In Fig. 6, the CV analysis highlights the distinctive behavior between CuS and CoS compared to CuCoS and CuCoS/PANI. Notably, for CuS and CoS, clear redox peaks are visible at lower scan rates, diminishing as the scan rate increases. This trend indicates their reduced stability in contrast to CuCoS and CuCoS/PANI, which maintain discernible redox peaks even at higher scan rates. This difference suggests a higher stability and sustained electrochemical activity in CuCoS-based materials.

From the CV curves, we are able to identify that the positions of the oxidation and reduction peaks moved to both sides as the scan rate increased. This might be caused by a limitation in the progress of OH^−^ ion diffusion in the redox process. The following assumptions can be assumed for the electrochemical redox reaction:

3 CoS + OH^−^ ↔ CoSOH + H_2_O + e^−^

4 CuS + OH^−^ ↔ CuSOH + H_2_O + e^−^

5 CuCoS + OH^−^ ↔ CuCoSOH + H_2_O + e^−^

This is due to the rapid reaction of electrolytes and electrodes. Fig. S3† demonstrates a strong linear connection between the maximum current densities and √v, providing more definitive proof that OH^−^ ion diffusion is the diffusion regulated mechanism.

The following formula derives specific capacity *via* CV analysis.^38^6
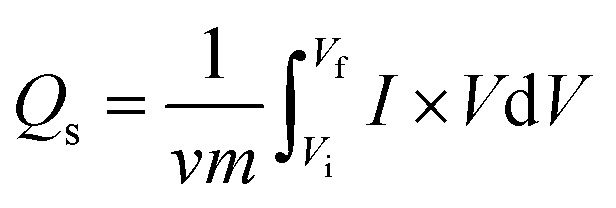


Fig. 8(a) shows the *C*_s_ computed for CuS, CoS, CuCoS, and CuCoS/PANI at various scan rates. The *C*_s_ values for CuS, CoS, CuCoS, and CuCoS/PANI at 3 mV s^−1^ were 401, 922, 1318, and 1509, respectively (Fig. 8(b)). The increased *C*_s_ with CuCoS/PANI can be attributed to higher SSA, large pore size and pore volume. A more synergetic impact makes the movement of ions faster. The ECSA was determined through CV measurements at a scan rate of 5 mV s^−1^. The capacitance has been calculated from the slope of the linear fit of plots of current density against scan rate.

The roughness factor (*R*_f_) of the working electrode must first be determined using the equation below in order to derive the ECSA.

7 ECSA = *R*_f_*S*

where *S* is typically equivalent to the electrode's geometric area (6.5 mg in this experiment). Using the relationship below, the *R*_f_ was calculated.8
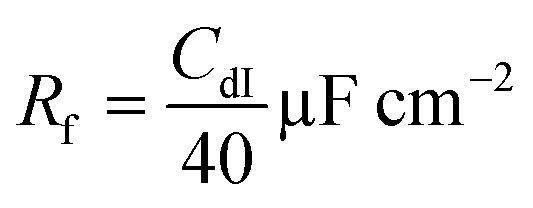


CuS, CoS, CuCoS, and CuCoS/PANI have computed ECSA values of 26, 31, 35, and 41.23 m^2^ g^−1^, respectively. Interestingly, these values agree well with the BET surface measurements, highlighting the dependability and stability of our results.

Galvanostatic charge/discharge (GCD) is an electrochemical approach for measuring the capacity and efficiency for electrode materials. GCD is the process of periodically charging and discharging a material or system within a particular potential range using a constant positive and negative current. GCD measurements are employed to determine the quality of capacitive reactions, recognize the potential for irreversible faradaic reactions, and deduce several essential electrochemical figures of merit, such as capacity, energy, and power. Advanced modeling approaches that can control the electric current are desirable in order to simulate the charging and discharging process.

**Fig. 7:**
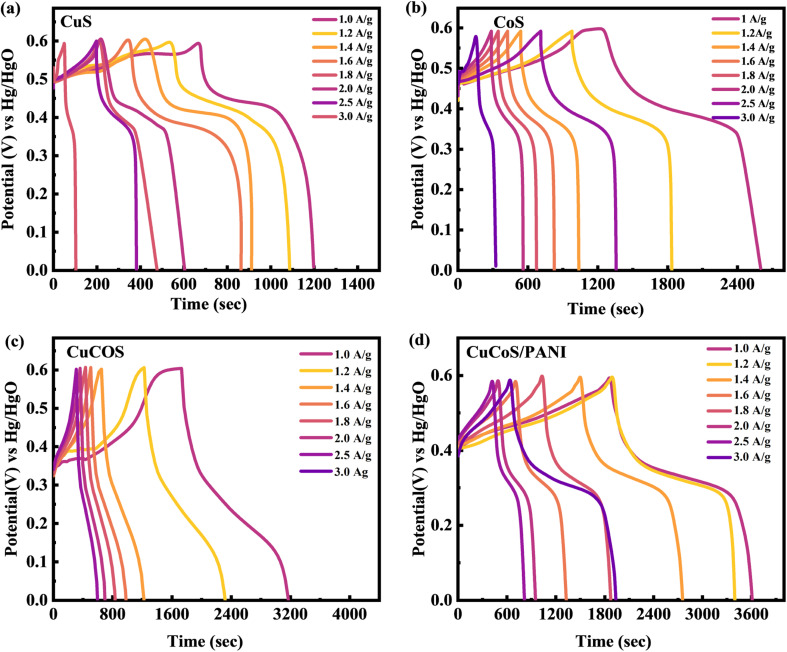
(a–d) GCD tests for CuS, CoS, CuCoS, and CuCoS/PANI, respectively.

GCD calculations were also performed for CuS, CoS, CuCoS and CuCoS/PANI in three distinct electrode assemblies (Fig. 7). The non-linear GCD curves indicated the presence of a redox reaction, which is consistent with the CV curves. The GCD profiles for CuCoS indicated a lower specific capacity, which was then increased by blending PANI with the CuCoS. In the GCD experiment, it became clear that the duration of charging/discharging becomes less at higher currents. The PANI nanofibers were cross-linked with one another in a perfectly distributed framework which resulted in a highly accessible interface. More redox-active sites were also constructed in the PANI to speed up the charge–discharge process. The non-linear shape of the GCD curves for CuS, CoS, CuCoS, and CuCoS/PANI indicates battery-graded performance. Faradaic responses related to battery dynamics are illustrated in peaks at different current densities. The GCD results showed that increasing the current density shortened the discharge time. At higher current densities, the discharge trajectory is shorter since less time is provided to the ions for interaction with the electrode.

The graphs indicate that CuCoS/PANI has a significantly greater specific capacity than the other samples because its discharge time is much higher. The specific capacity of a CuCoS electrode can be determined using the following equation:^48^9
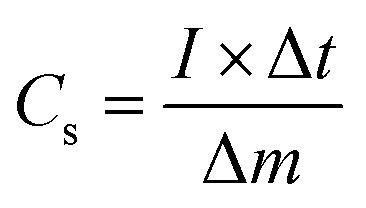


Specific capacity is denoted by *C*_s_ (C g^−1^), the applied current by *I*, the time by *t*, *V* is the potential, and the load of the working substance in the electrode by *m*.

**Fig. 8:**
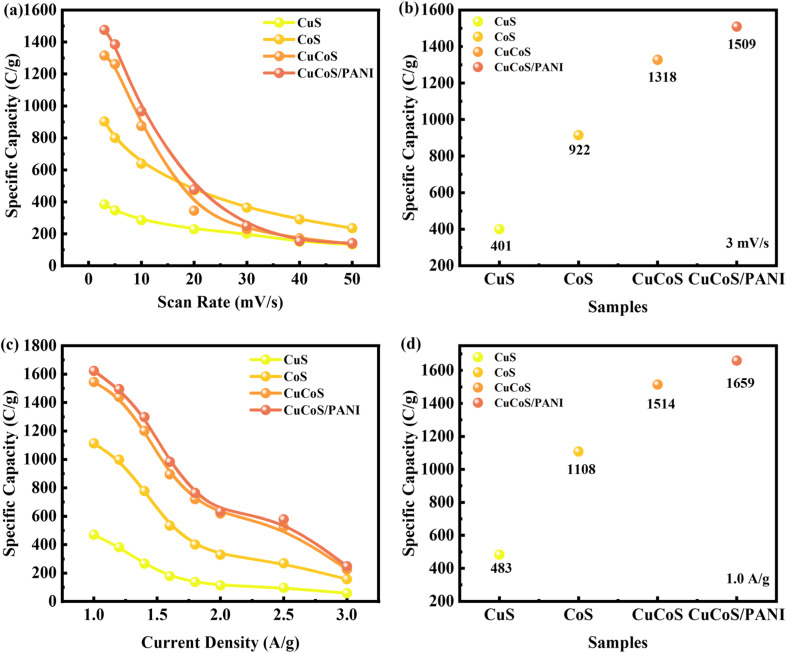
(a) *Q*_s_ computed through CV at various scans for CuS, CoS, and CuCoS/PANI. (b) *Q*_s_ computed through CV at 3 mV s^−1^ for CuS, CoS, and CuCoS/PANI. (c) *Q*_s_ computed through GCD at various current densities for CuS, CoS, and CuCoS/PANI. (d) *Q*_s_ computed through GCD at 1.0 A g^−1^ for CuS, CoS, and CuCoS/PANI.

The specific capacity at various current densities can be seen in Fig. 8(c). The specific capacity of samples decreases as the current density increases. This is because ions have excellent interactions with the electrode's electro-active regions at lower current density. Since electrolyte ions have poor diffusion at high current densities, an insufficient number of reaction sites are accessible, leading to an incomplete insertion reaction and a low specific capacity. The results present the CuCoS strong rate capability at high current densities. The *C*_s_ values for CuS, CoS, CuCoS, and CuCoS/PANI at 1.0 A g^−1^ were 483, 1108, 1514, and 1659, respectively (Fig. 8(d)). The specific capacitances are presented in Fig. S4.†

### 3.3. Hybrid device electrochemical performance

**Fig. 9:**
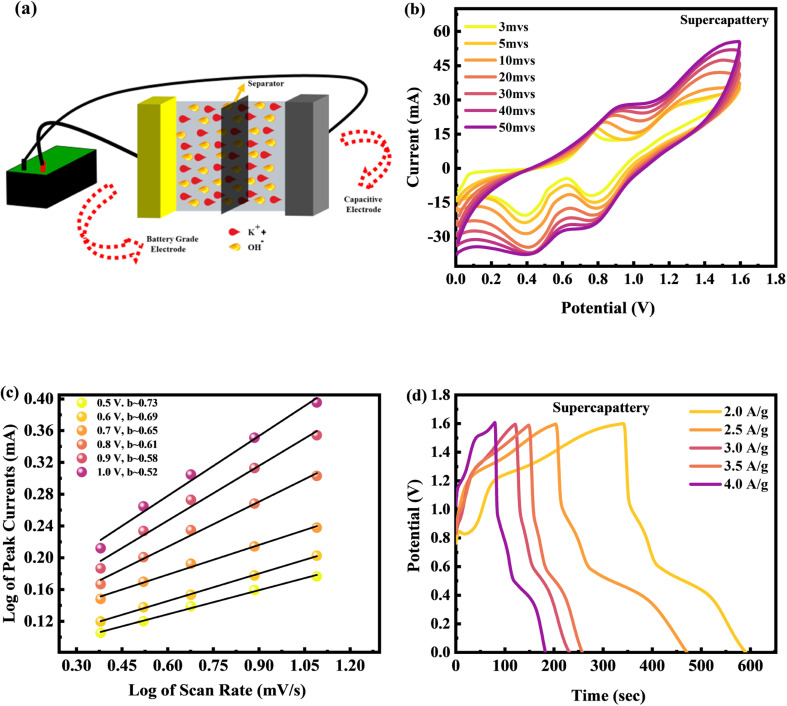
(a) Systematic approach to hybrid device design. (b) CV tests for the CuCoS/PANI//AC device at different scans. (c) *b*-Fitting for the CuCoS/PANI//AC device. (d) GCD tests for the CuCoS/PANI//AC device at different current densities.

The energy storage capacity is further investigated by developing a hybrid device. We assembled a hybrid device with CuCoS/PANI at the anode (battery grade material) and activated carbon (AC) at the cathode (capacitive material) for a real device application. Fig. 9(a) shows a schematic representation of the hybrid device. The electrical characteristics of CuCoS/PANI were investigated using a two-step construction process in a real device. A WHATMAN paper-based semipermeable membrane was implemented as a separator. A 2 M potassium hydroxide (KOH) solution in DIW was used as the electrolytic solution. Before preparing a hybrid device, both electrodes must be stable. Fig. 9(b) shows the cyclic voltammetry (CV) graphs of the hybrid device. The range of the operational potential (O.P.) in a two-electrode setup was from 0 to 1.6 V. By using cyclic voltammetry with several scanning rates (at 3, 5, 10, 20, 30, 40, and 50 mV s^−1^), the rate capability and stability of the CuCoS/PANI//AC asymmetric device were checked. The CV curves exhibited a rectangular shape indicative of capacity at low potentials (0 to 0.5) which is caused by the electrolyte adsorption of ions. The faradaic reaction contributes to charge storage at high potentials (>0.5), demonstrated by redox peaks. This demonstrated that the device had the characteristics of both a capacitor and a battery. Overall, these CV plots show that the device exhibits both capacitive and battery-type behaviour, indicating the formation of a hybrid device. The CuCoS/PANI//AC asymmetric device revealed high rate capability and stability, even at higher scanning speeds.

The relationship between scan rate (*v*) and current (*i*) implies the power law which was used to distinguish the charge storage mechanism.

10 *i* = *av*^*b*^

Here, *a* and *b* are two parameters that may be changed.

The *b*-fitting value is shown in Fig. 9(c), plotted along the potential. The results indicate that the *b*-values are between 0.52 and 0.73. These *b*-values are significant because they distinguish between various forms of energy storage devices.

A battery-operated device is indicated by a *b*-value between 0 and 0.5. *b*-Values between 0.5 and 0.8 are indicative of a hybrid device, whereas values of *b* more than 1.0 are typical of supercapacitors.^48^ These *b*-values are consistent with theoretical expectations, lending credence to our assertion about the composition of the energy storage systems.

The GCD measurements for the device are carried out in a two-electrode setup with a potential window (PW) of 0–1.6 V at various currents (1.0–3.0 A g^−1^) with 2 M KOH as the electrolyte. The discharging results of the device are shown in Fig. 9(d). These GCD curves exhibit both capacitive and battery-like behaviours, as seen by the small humps, in support of our hypothesis of hybrid device development.

**Fig. 10:**
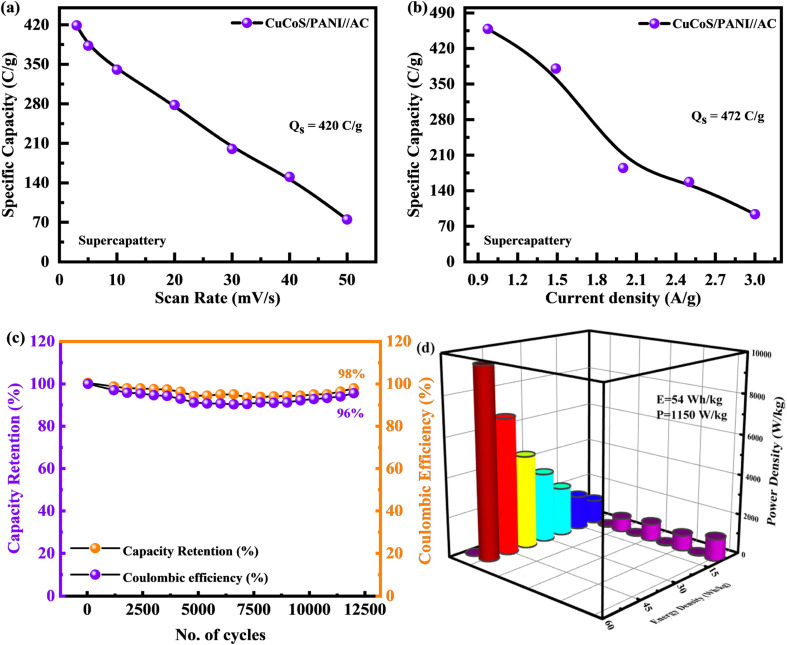
(a and b) *Q*_s_ computed through CV and GCD for CuCoS/PANI//AC. (c) Stability test for the CuCoS/PANI//AC device after 12 500 GCD cycles. (d) Comparison of *E*_d_ and *P*_d_ for the CuCoS/PANI//AC device with the literature.

The *C*_s_ of the CuCoS/PANI//AC device was calculated from both CV and GCD tests and presented in Fig. 10(a) and (b). The CuCoS/PANI//AC device showed *C*_s_ of 420 C g^−1^ at 3 mV s^−1^ and 472 C g^−1^ at 2.0 A g^−1^.

As can be seen in Fig. 10(c), the device had a very long lifetime stability. The lifetime of this hybrid device (CuCoS/PANI//AC) was determined by exposing it to 12 500 charging–discharging cycles at 5.0 A g^−1^. After 12 500 continuous GCD cycles, the device maintains its capacity retention at 96% and coulombic efficiency of 98%. The modest increase in particular capacity after 1000 cycles indicates that the electrodes had stabilized at their maximum capacity. Electrolyte ion transport into the active material improves with increasing cycle number, resulting in gradually increased redox activity. As a result, the battery capacity of the devices will expand. Table 2 shows the comparison for the CuCoS/PANI//AC device performance with the literature.

**Table 2:** Comparison of the coulombic efficiency and capacity retention of the CuCoS/PANI//AC device with the literature

Previous literature	No. of cycles	Coulombic efficiency (%)	Capacity retention (%)	Ref.
Manganese oxide	400	85	74	53
Na_4_Mn_9_O_18_/Na_2_SO_4_/AC	4000	85	84	54
Zinc phosphate–rGO	2000	106	71	55
Ni/Co-MOFs	5000	98.4	73	56
TiO_2_@rGO	10 000	83%	80%	57
CoO urchin-like microspheres	5000	—	84.65%	58
SrCo_0.9_Fe_0.1_O_3−*δ*_@CC//AC@CC	5000	—	85.71%	59
**CuCoS/PANI//AC**	**12 500**	**98%**	**96%**	**This work**

The energy storage capability, such as specific energy and specific power, is determined using the following equations:^49,50^11
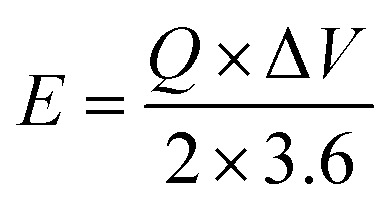
12
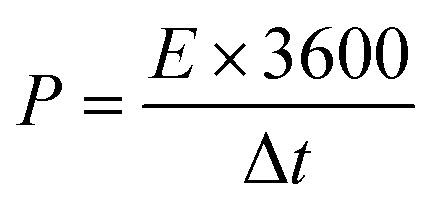


The hybrid device has a high specific energy of 54 W h kg^−1^, at a specific power of 1150 W kg^−1^. The maximum power of 10 896 W kg^−1^ was achieved at 14 A g^−1^ energy. In terms of specific energy and power, Fig. 10(d) illustrates a comparison between device performance and previously published literature. Table 3 was also made for comparison.

**Table 3:** Comparison of *E*_d_ and *P*_d_ for CuCoS//PANI with previous work

Previous study	Energy density	Power density	Reference
CoS_2_/CuCo_2_S_4_‖N–rGO	32.4 W h kg^−1^	4000 W kg^−1^	60
Cu_2_(PO_4_)(OH)	3.85 W h kg^−1^	264.70 W kg^−1^	61
Cu_2_S microspheres	25.4 W h kg^−1^	4.1 kW kg^−1^	62
AgCuS core/shell	10.01 W h kg^−1^	520 W kg^−1^	63
Ni_3_S_2_@CoS	28.2 W h kg^−1^	0.13 kW kg^−1^	64
SrFeO_3−*δ*_ perovskites	16.9 W h kg^−1^	984 kW kg^−1^	65
Cu_2_MoS_4_	16.8 W h kg^−1^	800 kW kg^−1^	66
Fe_3_Mo_3_C/Mo_2_C@carbon nanotubes	39.9 W h kg^−1^	1800 kW kg^−1^	67
**CuCoS/PANI**	**54 W h kg^−^** ^**1**^	**1150 W kg^−^** ^**1**^	**This work**

**Fig. 11:**
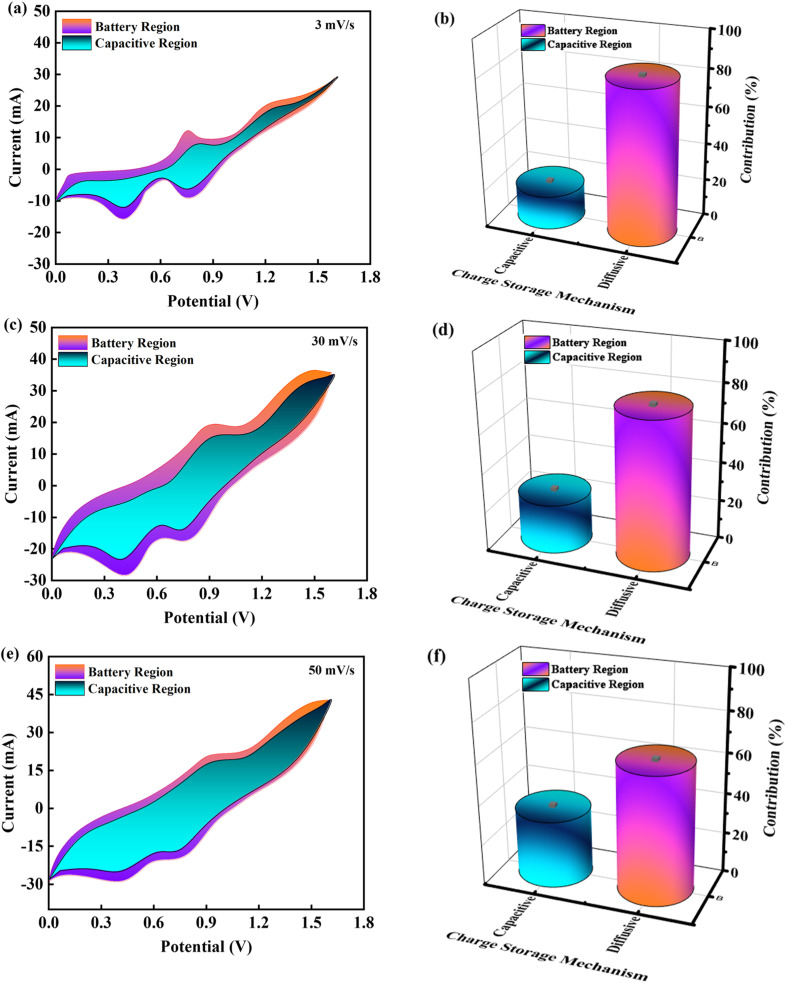
Dunn's calculations of capacitive and diffusive contributions at (a) 3 mV s^−1^, (c) 30 mV s^−1^, and (e) 50 mV s^−1^. Bar chart showing capacitive and diffusive contributions at (b) 3 mV s^−1^, (d) 30 mV s^−1^, and (f) 50 mV s^−1^.

Two CuCoS–PANI//AC devices were connected in a hybrid configuration to demonstrate their increased functionality (Fig. S5†). Dunn's model might be simply used to analyze the predominant technique of storing charges in electrochemical processes (Fig. 11). The capacitive and diffusive components of the total capacity of the devices can be measured quantitatively. Current contribution is shown as the sum of capacitive and diffusive currents, which provides insight into charge kinetics.

13 *i*(*v*) = *i*_capacitive_ + *i*_diffusive_

In eqn (11), the output current for a specific potential is denoted by *i*(*V*), and *v* represents the scan rate. The following equation is used to distinguish the capacitive and diffusive parts.

14 *i*(*V*) = *k*_1_*v* + *k*_2_*v*^1/2^

where *k*_1_*v* represents the surface capacitive component and *k*_2_*v*^1/2^ represents the diffusive component.

Fig. 11(a)–(f) show the statistical results at several scan rates to clearly distinguish between currents generated by diffusion and adsorption processes.

The capacitive contribution to the total capacity of the hybrid device is found to be 19% at a scan rate of 3 mV s^−1^, the diffusive contribution seems to be dominant due to the ions having enough time to abound in the redox relations, allowing charge storage with the assistance of a battery-grade electrode.

As can be seen in the bar plot of Fig. 11(d) and (f), the capacitive contribution rises with increasing scan rate. This is because the capacitive electrode charge storage contribution becomes significant at high scan rates, preventing the ions from having enough time to complete the adsorption/desorption process. Both capacitive and battery-grade electrodes contribute to charge storage, as seen above, confirming the device's hybrid nature.

### 3.4. Oxygen evolution reaction (OER)

Tafel slopes and linear scan voltammetry (LSV) plots in 2 M KOH at a scan rate of 3 mV s^−1^ were used to evaluate the OER catalytic performance of the catalysts. All OER potentials were reported with respect to a reversible hydrogen electrode (RHE). The electrocatalysts CuCoS and CuCoS/PANI for the oxygen evolution reaction in water electrolysis were investigated in this experiment in comparison to the commercially available IrO_2_ catalyst. Using the Nernst equation, the calculated potentials were converted from the SCE scale to the RHE scale.15
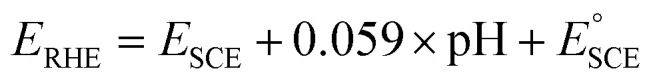
*E*_RHE_ is the reverse potential and *E*_SCE_ is the experimental voltage across the standard reference electrode (SRE). 
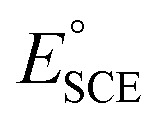
 is the SCE redox potential at 297 K (0.234 V, 0.002 V) according to the standard. Using the equation, the LSV curve was used to figure out the overpotential, *η*, for a given current density.

The electrode catalytic efficiency can be estimated by calculating the overpotential to produce a current density of 10 mA cm^−2^. Moreover, the lower overpotential predicts a significantly higher performance towards the OER.

16 *η* = *E*_RHE_ − 1.23 V

The Tafel plot demonstrates the significance of catalytic kinetic analysis as a determinant of the OER. Eqn (15) was used to calculate the sample Tafel slopes.^51^

**Fig. 12:**
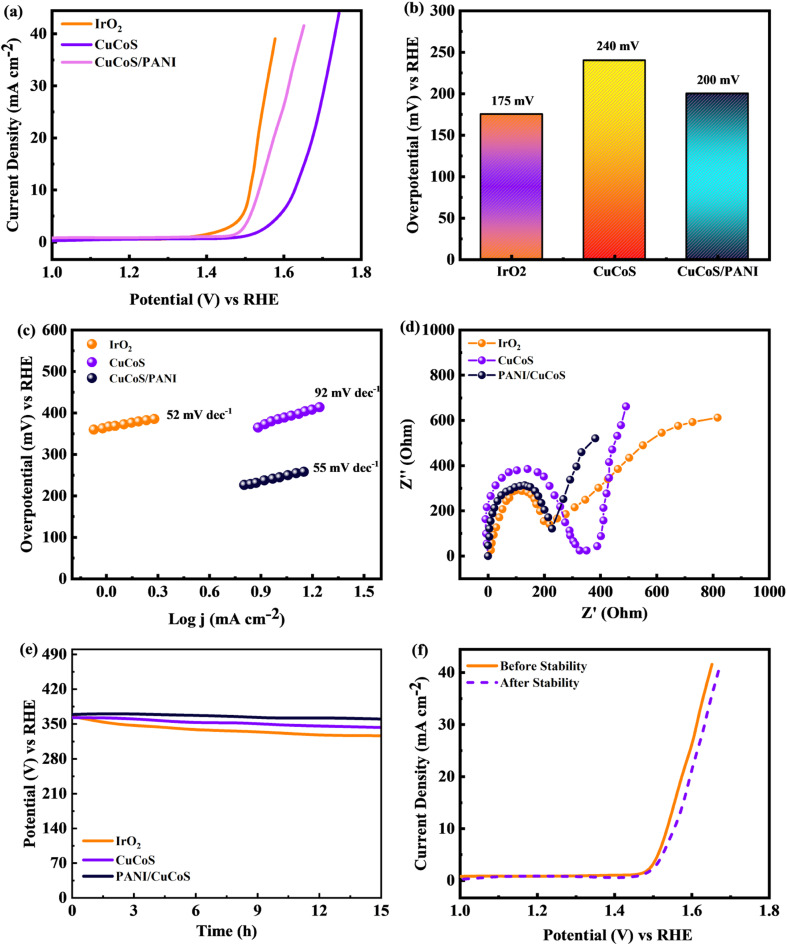
(a) LSV curves for IrO_2_, CuCoS, and CuCoS/PANI. (b) Bar chart showing the overpotential required in HER applications for IrO_2_, CuCoS, and CuCoS/PANI. (c) Tafel slope measurements for IrO_2_, CuCoS, and CuCoS/PANI. (d) EIS plots for IrO_2_, CuCoS, and CuCoS/PANI. (e) Stability test for IrO_2_, CuCoS, and CuCoS/PANI after 15 h. (f) LSV curve for CuCoS/PANI before and after the stability test.

17 *E* = *b* log *J* + *a*

where *E* is the overpotential, *J* is the current density, *a* is a constant, and *b* is the Tafel slope. Fig. 12(a) shows the LSV curves for CuCoS, CuCoS/PANI, and IrO_2_. The initial overpotential of the CuCoS film is 240 mV. The CuCoS/PANI hybrids enhanced OER activity compared to CuCoS, as seen in Fig. 12(b). The improved CuCoS/PANI hybrid obtained the lowest overpotential of 200 mV at 10 mA cm^−2^ which was close to that of IrO_2_ (175 mV). The OER performance of CuCoS/PANI is significantly higher than that of previously identified Co-based catalysts. In Fig. 12(c), the OER kinetics were analyzed using Tafel plots. The optimized hybrid had a much lower Tafel slope (55 mV dec^−1^) as compared to CuCoS (92 mV dec^−1^). The obtained Tafel slope was very close to that of the benchmark IrO_2_ catalyst (52 mV dec^−1^). A less steep Tafel plot or a smaller Tafel slope signifies better electrocatalytic activity or lower energy requirements for the oxygen evolution process.^52^ In a three-electrode setup, Fig. 12(d) shows the electrochemical impedance spectra (EIS) for both CuCoS and CuCoS/PANI hybrids. The solution resistance (*R*_s_) was approximately 1.5 Ω for the CuCoS/PANI material which was lower than that of CuCoS. The electrocatalytic activity for the OER increases with decreasing *R*_ct_, as is the case with LSVs and Tafel electrodes. Thus, it is determined that more rapid catalytic kinetics contributed to the improved activity of CuCoS/PANI. The improved hybrid OER stability was measured for future investigations. Table 4 was made for comparison of the Tafel slope and overpotential for the CuCoS/PANI device with the literature.

**Table 4:** Comparison of the overpotential and Tafel slope of CuCoS/PANI with the literature

Material	Overpotential	Tafel slope	Reference
Co_9_S_8_	217 mV @ 10 mA cm^−2^	110 mV dec^−1^	68
CoS	212 mV @ 10 mA cm^−2^	98 mV dec^−1^	69
CoMoS_4_	143 mV @ 10 mA cm^−2^	105 mV dec^−1^	70
Co_0.75_Ni_0.25_Se	106 mV @ 10 mA cm^−2^	32 mV dec^−1^	71
CoS_2_/CC	291 mV @ 10 mA cm^−2^	67 mV dec^−1^	72
CoCuFe–S-8	79 mV @ 10 mA cm^−2^	431 mV dec^−1^	73
Co_3_O_4_/MoS_2_	230 mV @ 20 mA cm^−2^	45 mV dec^−1^	74
**CuCoS/PANI**	**200 mV @ 10 mA cm^−^** ^**2**^	**55 mV dec** ^**−1**^	**This work**

Fig. 12(e) shows that the OER activity of the CuCoS/PANI remained constant and that no noticeable potential increase was observed for more than 15 h of oxygen release. The stability was also evaluated by taking the LSV plot for CuCoS/PANI before and after 5000 CV measurements. It can be observed in Fig. 12(f) that there was a small increase in overpotential after the stability test. All of the above discussion indicates that CuCoS/PANI has great potential in future energy storage devices and oxygen evolution reactions.

## 4. Conclusion

In this study, CuS, CoS, CuCoS, and CuCoS/PANI materials were effectively hydrothermally synthesized and used as electrodes in supercapacitors. One of them, CuCoS/PANI, showed exceptional performance in a three-cell design with 1659 C g^−1^ capacity. The substance is a great choice for energy storage applications due to its amazing flexibility and resistance to extreme stress. The asymmetric supercapacitor produced an exceptional energy density of 54 W h kg^−1^ at 1150 W kg^−1^ power density using activated carbon as the cathode and CuCoS/PANI as the anode. Additionally, the device showed outstanding stability, maintaining 98% coulombic efficiency and 96% of its original capacity after 12 500 GCD cycles. The research also demonstrated the potential of CuCoS/PANI as an electrocatalyst for the oxygen evolution process, with a 55 mV dec^−1^ Tafel inclination and 200 mV overpotential. CuCoS/PANI stands out as a viable electrode material for energy storage devices and as an effective electrocatalyst for oxygen evolution processes due to its high electrochemical performance, flexibility, and stability. This study paves the way for CuCoS/PANI to play a significant role in determining the direction of energy storage and electrocatalysis technologies in the future.

## Data availability

All data underlying the results are available as part of the article and no additional source data are required.

## Conflicts of interest

There are no conflicts to declare.

## Supplementary Material

NA-006-D3NA01066J-s001
