# Supplementary material for: Synergistic CuCoS–PANI materials for binder-free electrodes in asymmetric supercapacitors and oxygen evolution
Source: Nanoscale Adv. 2024 Jan 26;6(5):1507–23. doi: 10.1039/d3na01066j (PMC10898445; doi:10.1039/d3na01066j)
Supplement: NA-006-D3NA01066J-s001 [file NA-006-D3NA01066J-s001.pdf]

Electronic Supplementary Material (ESI) for Nanoscale Advances.
This journal is © The Royal Society of Chemistry 2024

Synergistic CuCoS-PANI Materials for Binder-Free Electrodes in Asymmetric Supercapacitors and Oxygen Evolution

Haseebul Hassan[1], Muhammad Waqas Iqbal[1]*, Hussein Alrobei [2], Fareeha Riasat[1], Amir Afzal[1], Ahmad M. Saeedi [3], Hasan B. Albargi[4], Arslan Rehmat[5]

[1]Department of Physics, Riphah International University, Campus Lahore, Pakistan

[2] Department of Mechanical Engineering, College of Engineering, Prince Sattam bin Abdul Aziz University, Al- Kharj, 11942, Saudi Arabia [3]Department of Physics, Faculty of Applied Science, Umm AL-Qura University, Makkah 24382, Saudi Arabia

[4]Department of Physics, Faculty of Science and Arts, Najran University, PO Box 1988, Najran, 11001, Saudi Arabia

[5]Department of Physics, Sejong University, South Korea

E-mail: waqas.iqbal@riphah.edu.pk,

Supplementary Section:

1.1. Electrode Conductivity Measurements:

Below equations were applied for conductivity measurements [36].

$$\rho = \frac{\rho_0}{G_7}(\frac{W}{S}) \qquad (7)$$

$$G_7\left(\frac{W}{S}\right) = \left(\frac{2S}{W}\right)\ln 2 \tag{8}$$

$$\rho_o = \left(\frac{V}{I}\right)2\pi S \tag{9}$$

$$\sigma = \frac{1}{\rho_o} \tag{10}$$

The conductivity tests for CuS, CoS, CuCoS, and CuCoS/PANI are shown in Figure S1(a). CuS conductivity ranged from 0.12 to 6.28 S/m. Similarly, CoS has conductivity ranging from 0.35 to 12.42 S/m. CuCoS, a composite material, demonstrated conductivity ranging from 1.23 to 22.63 S/m. The total conductivity range of the CuCoS-PANI composite was 1.56-27.81 S/m.

Electrolyte Conductivity Test

Potassium hydroxide (KOH) solutions with varied molarities of 0.5, 1, 1.5, 2, 2.5, and 3 M were used to test the electrolyte conductivity (Figure S1b). Using a conductivity meter set up to precisely measure each KOH solution's electrical conductivity was part of the conductivity measuring procedure. The meter precisely determined each KOH solution's conductive characteristics by submerging conductivity probes in it. The purpose of employing a variety of molarities was to methodically examine the connection between conductivity (κ) and electrolyte concentration.

$$\kappa = K_{Cell}G \tag{11}$$

In this case, the terms K_{cell} and G denote the cell's conductivity and conductance, respectively, which are obtained from the plate spacing to area (d/A) ratio. Variations in temperature and the concentration of the electrolytic solution have a significant impact on the electrical conductivity of the electrolyte. The technique combined a number of precise equipment, including a

conductivity gauge, a temperature-controlled water bathtubs, conductivity cells, digitized thermometers, and precision measurement vessels, in accordance with the methods described by R. Blaine McCleskey et al. [37]. It was easier to get conductivity readings throughout the designated molarities by using a ratiometric resistance approach with both known and unidentified resistances linked to an AC source. The resultant results, which are shown graphically in Figure 2(b), showed a clear pattern emphasizing how molarity affects KOH conductivity. Interestingly, from 0.5 to 1.5 M, the conductivity showed an upward trajectory, indicating that conductivity increased as the electrolyte solution's molarity increased. The maximum 0.57 S/m conductivity was achieved with 2 M KOH electrolyte. This rise is associated with an increase in the concentration of ions in the solution, which increases conductivity by making more charge carriers accessible for current transfer. Beyond 2 M, a divergence from this pattern was noted, with additional molarity increases leading to a decrease in conductivity. Beyond 2 M, conductivity often decreases and is typically linked to ion interactions that are frequent in concentrated solutions. Ions have a tendency to form complexes or interact at larger concentrations, which limits ion mobility and prevents efficient electrical conduction. These results are consistent with previous research and demonstrate the complex connection between the amount of ions and subsequent conductivity developments.

In this part diffusive participation were also evaluated from CV charts. Figure S2 represents the SEM images for CuS and CoS. Figure S3 demonstrates a strong linear connection between peak current densities and the square roots of the scan rates, providing more definitive proof that OH^- ion diffusion is the diffusion regulated mechanism. These peaks provide information on the characteristics of the faradic reaction. If the peaks of the oxidation and reduction peaks overlap, the reaction is reversible; otherwise, it is quasi-reversible, and if both peaks diverge, the reaction is irreversible. The battery-graded nature of the CV urves was further demonstrated by deviations

from the characteristic rectangular shape. The anodic and cathodic peaks shift to higher and lower potentials, respectively, as the scan rate increases.

Randles Sevciks concept applies to more diffusion-controlled processes. CuS, CoS, CuCoS and CuCoS/PANI CV curves were plotted along the square root of the scan to determine the anodic and cathodic peak currents, respectively. This linear relationship between scan rate and peak currents demonstrates the overall performance of battery-type materials. Anodic and cathodic currents rise with increasing scan rate. As can be seen in Figure 3(a-d), the R^2 value was also calculated independently for CoS, CuS, CuCoS and CuCoS/PANI. The R^2 value for battery-type material is quite near to 1. The battery-like behavior of CoS, CuS, CuCoS and CuCoS/PANI is further demonstrated by their R^2 values, which range from 0.90 to 0.99 when measured by anodic and cathodic currents, respectively.

$$i_p = 0.4463nF\sqrt{\frac{nFD}{RT}}AC\sqrt{V}$$

Ip represents peak current, n indicates the number of electrons, T for temperature, F indicates the faraday constant, D stands for the diffraction coefficient, A for the area, C for the bulk concentration. Using this formula, peak currents were calculated and plotted versus the square root of the scan.

(Figure S3(a-d)). It can be determined that diffusion-controlled processes are primarily responsible for charge storage because of the linear relationship between the square root of the scan and peak currents. The specific capacitance were also calculated by dividing the specific capacity with operating potential. Figure S4(a) represents the specific capacitance computed through CV at various scan rate. The specific capacitance for CuS, CoS, CuCoS, and CuCoS/PANI were 668, 1536, 2196, 2515 F/g (Figure S4b). The specific capacitance were also computed

through GCD at different current densities were plotted in Figure S4(c). The specific capacitance for CuS, CoS, CuCoS, and CuCoS/PANI were 805, 1846, 2523, 2765 F/g (Figure S4(d).

This effect was illustrated in detail in Figure S5(a), where interesting results were obtained from cyclic voltammetry (CV) measurements at 20 mV/s for just one and dual CuCoS-PANI//AC devices linked in series. The operational range (OP) of the single device was 0–1.6 V, but when two devices were coupled, the OP increased to an astounding 0–3.2 V, significantly expanding the operating spectrum. Fascinatingly, the CV curve shapes for both setups were similar, with a rectangular profile and small redox spikes. These characteristics are suggestive of charge storage processes that involve faradaic reactions and adsorption/desorption.

Furthermore, the GCD tests, which show a linear increase in OP with an increased device count, confirmed this pattern in Figure S5(b). The symmetric triangle pattern with minimal internal resistance drop was demonstrated by the GCD curves, which held their original shape even after two CuCoS-PANI//AC devices were integrated in series. This finding highlights the dual setup's potential for reliable energy storage applications. These results highlight the adaptability and potential to improve energy storage capabilities of CuCoS-PANI//AC configurations, underscoring their promise for use in future energy storage technologies.

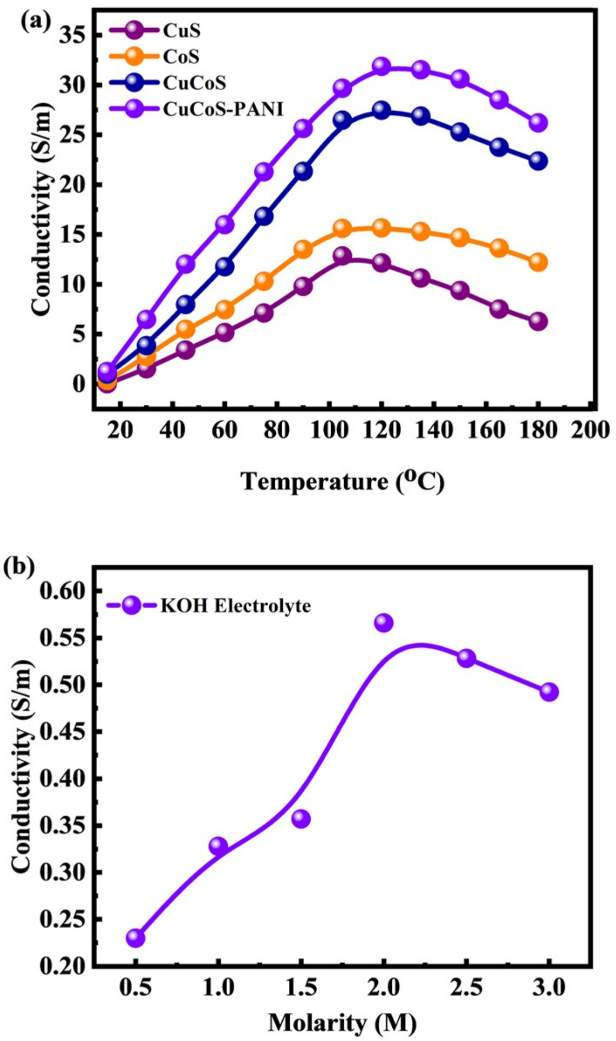

Figure S1. Conductivity measurements for (a) electrodes and (b) electrolytes

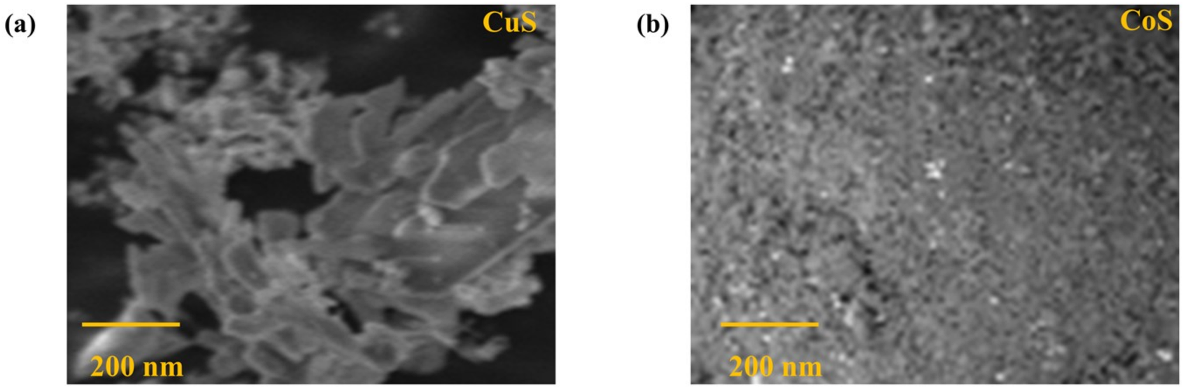

Figure S2. (a-b) SEM images for CuS and CoS.

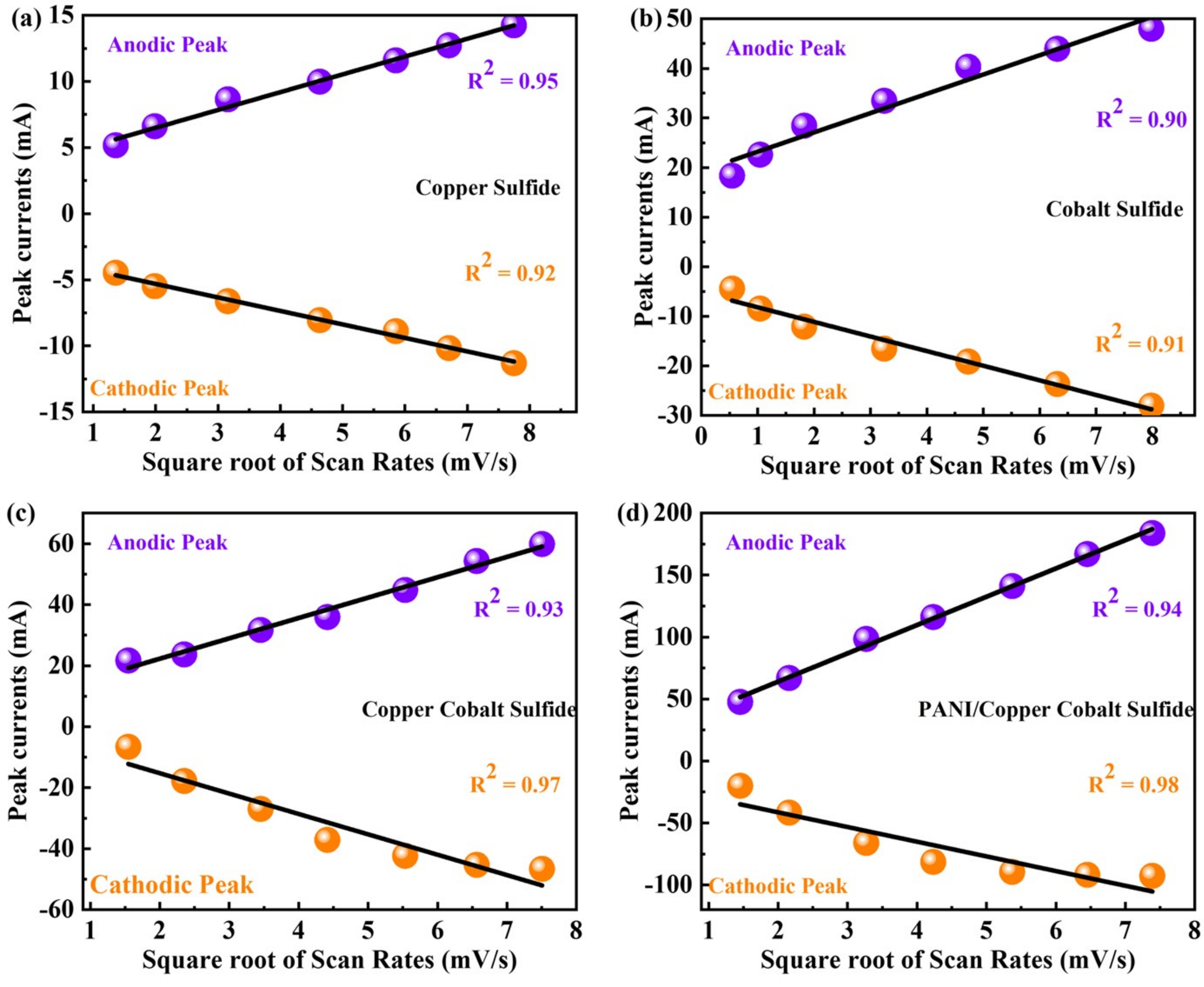

Figure S3. (a-d) Randles Sevcik's model applied on CuS, CoS, CuCoS, and CuCoS/PANI, respectively.

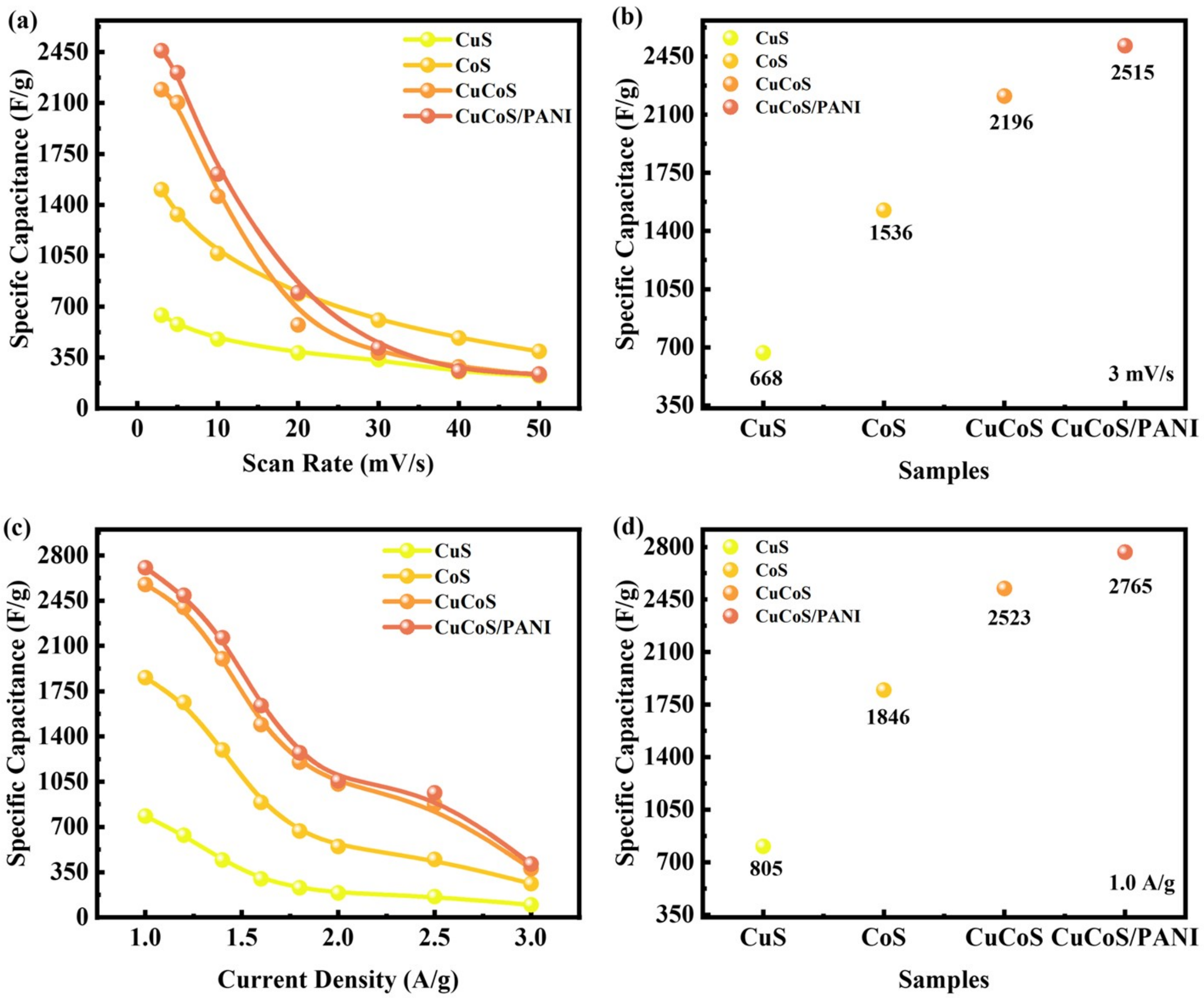

Figure S4. (a) Specific capacitance computed through CV at various scan rate. (b) Specific capacitance computed at 3 mV/s for CuS, CoS, CuCoS, and CuCoS/PANI. (c) Specific capacitance computed through GCD at various current densites. (b) Specific capacitance computed at 3 mV/s for CuS, CoS, CuCoS, and CuCoS/PANI.

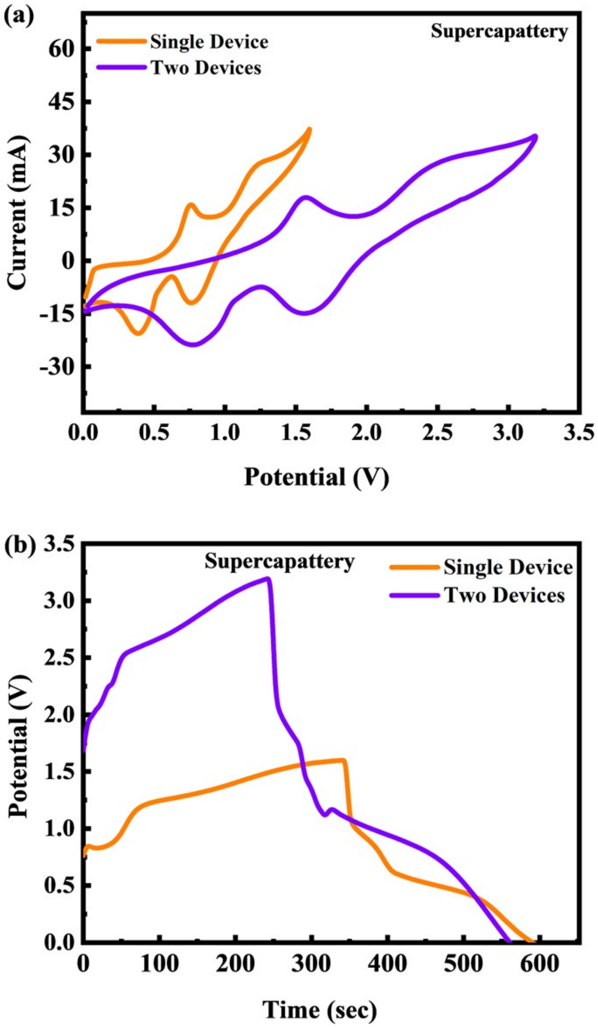

Figure S5. Comparison of single and two device performance (a) through CV and (b) through GCD.
